# Group intervention for family members of people with borderline personality disorder based on Dialectical Behavior Therapy: Implementation of the Family Connections® program in France and Switzerland

**DOI:** 10.1186/s40479-024-00254-3

**Published:** 2024-07-23

**Authors:** Satchel Cohen, Virginie Salamin, Nader Perroud, Karen Dieben, Déborah Ducasse, Amaury Durpoix, Florence Guenot, Hervé Tissot, Ueli Kramer, Mario Speranza

**Affiliations:** 1 Child and Adolescent Psychiatry Care Unit, Versailles Hospital, Le Chesnay, France; 2 Réseau Fribourgeois de Santé Mentale, Fribourg, Switzerland; 3 Department of Psychiatric Specialties for Emotion Regulation Disorders, Geneva University Hospitals, 1201 Geneva, Switzerland; 4 CHU de Montpellier, Service Urgences Et Post-Urgences Psychiatriques (Lapeyronie), Centre de Thérapies Troubles de L’humeur Et Émotionnels/Borderline (La Colombière), IGF, Univ. Montpellier, CNRS, Inserm, Montpellier, France; 5 Strasbourg University Hospital, 67000 Strasbourg, France; 6 Center for Family Studies, University Institute of Psychotherapy, Department of Psychiatry, Lausanne University Hospital and University of Lausanne, Lausanne, Switzerland; 7 Faculty of Psychology and Educational Sciences, University of Geneva, Geneva, Switzerland; 8 Lausanne University Hospital Institute of Psychotherapy/General Psychiatry, 1003 Lausanne, Switzerland; 9 Paris-Saclay University, UVSQ, Inserm, Centre for Research in Epidemiology and Population Health UMR 1018, Team “Developmental Psychiatry and Trajectories”, 78000 Versailles, France

**Keywords:** Family caregivers/education, Caregiver burden, Borderline personality disorder, Dialectical behavior therapy

## Abstract

**Background:**

Families and significant others of people with borderline personality disorder (BPD) show increased levels of psychological distress. Family Connections®, a 12-week group intervention based on the principles of Dialectical Behavior Therapy, was designed to provide families with both information about the disorder and emotion regulation skills. It has been progressively implemented in French-speaking European countries.

**Methods:**

We conducted an observational, multicenter study in France and Switzerland. In total, 149 participants of the Family Connections program were included among five centers. Burden, depression, coping, and emotion regulation were assessed before and after the intervention.

**Results:**

One-way repeated measures MANOVA showed that the burden, depressive symptoms, emotion regulation and coping all changed significantly after the intervention (*p* < 0.001, partial η^2^ = 0.297). T-tests showed that the burden significantly decreased after the intervention (*p* < 0.0001, d = -0.48), as did depressive symptoms (*p* < 0.0001, d = -0.36) and difficulties in emotion regulation (*p* < 0.0001, d =-0.32) whereas coping improved (*p* < 0.0001, d = 0.53). Two-way mixed ANOVA showed that burden reduction was stronger among female than male participants (*p* = 0.048, η^2^ = 0.027). Before the intervention, the burden was higher for female than male participants (*p* < 0.001). An initial linear regression showed the burden reduction to be associated with a decrease in the resignation of the participants (β = 0.19, *p* = 0.047). A second linear regression showed the burden reduction to be associated with the intensity of the relatives’ symptoms at baseline (β = 0.22, *p* = 0.008) and improvement of emotional clarity of the participants (β = 0.25, *p* = 0.006).

**Conclusion:**

This Dialectical Behavior Therapy-Based psychoeducational intervention is an appropriate way to support French-speaking European families of people with BPD.

**Supplementary Information:**

The online version contains supplementary material available at 10.1186/s40479-024-00254-3.

## Background

Borderline personality disorder (BPD) is characterized by a pervasive pattern of instability in affect regulation, impulse control, interpersonal relationships, and self-image [1, 2]. Community-based studies have reported a prevalence of 0.7 to 2.7%, whereas in clinical settings, the disorder concerns up to 22.4% of adults [3]. Dialectical Behavior Therapy (DBT) is among the most empirically-validated interventions for BPD [4, 5].

DBT is based on the principles of cognitive and behavioral therapy, dialectical thinking, and the practice of mindfulness. It focuses on skills training concerning tolerance to distress, emotion regulation, interpersonal effectiveness, and the practice of mindfulness [6]. The DBT framework posits a developmental model of BPD, with a focus on the role of early family interpersonal interactions [7, 8]. According to the biosocial theory of Marsha Linehan, BPD is defined as an emotion dysregulation disorder that develops within an invalidating social environment [7]. Invalidation occurs through intolerance toward the expression of private emotional experiences, in particular when not supported by observable events [8]. In DBT, interpersonal validation thus becomes a key component of psychotherapy [9].

Extending Linehan’s theory, Fruzzetti, Shenk, and Hoffman elaborated a family transactional model of the development and maintenance of BPD [10]. In this model, the person with BPD and their family members reinforce each other’s emotion dysregulation and problematic behavior through invalidating transactions. The emotionally vulnerable person is more likely to behave in ways that reinforce the use of invalidating responses from family members. In turn, invalidating responses reinforce problematic behavior from the person with BPD. It emphasizes the need for specific work on transactions within the family system to improve emotion regulation.

Quantitative research has shown that the families of people with BPD experience high levels of psychological distress [11–14]. Qualitative research has highlighted the daily challenges for such families, such as dealing with stigma and a persistent feeling of threat [15, 16]. Moreover, this high psychological burden is associated with significant financial costs [17]. Family members also report feeling insufficiently involved in institutional care and struggling to obtain clear information [18]. They express their need for a better understanding of the disorder to adequately help their relatives with BPD [15, 16, 19, 20], as well as develop day-to day coping strategies for their own well-being [19–21].

Based on the transactional model, Fruzzetti and colleagues designed the Family Connections® (FC) program, a specific intervention for families of people with BPD. The program is grounded both in the DBT framework and in the stress and coping adaptation model by Lazarus and Folkman, in which the use of coping strategies are thought to be key resources in managing stressful situations [22]. It consists of a 12-week group intervention for families of people with BPD that aims to address the need of family members for information on the illness, develop coping strategies, and build a support network [23, 24]. Emotion regulation and interpersonal validation skills are particularly emphasized [9].

The FC program has been empirically evaluated by several studies. Two uncontrolled pre/post studies with follow-up measurements were first published in 2005 (*n* = 44) [23] and 2007 (*n* = 55) [24]. Measures of burden, depression, grief, and mastery were chosen to enable comparison with research studies undertaken on family members of people with mental illnesses other than BPD. Both studies showed a decrease in burden and grief scores after completion and an increase in mastery. One study, performed on a larger sample [24], also showed a decrease in depressive symptoms.

To date, only one non-randomized controlled study on the program that compared the FC program (*n* = 51) to treatment as usual (*n* = 29) has been published [25]. It also showed a decrease in burden, grief, and depression, and an increase in mastery after completion. The effect of the intervention was significantly superior to that of treatment as usual for burden and grief. One study compared the traditional weekly setting (FC-S, *n* = 34) with a shorter setting of two full-day weekend sessions (FC-R, *n* = 48) with follow-up measurements [26]. The authors evaluated global psychological suffering, family burden, quality of family functioning, quality of life, and mindfulness skills. No differences were found between groups in terms of global psychological suffering reduction and of quality of family functioning improvement. A difference between groups was found in terms of burden reduction, mainly due to a higher level of pre-intervention burden in the FC-S group. The quality of life and mindfulness skills did not increase over time.

Finally, three studies evaluated the FC program for family members of people experiencing a larger range of symptoms than only those of BPD. One pre/post study evaluated the program adapted for families of those who attempted suicide (*n* = 13) [27]. The results showed a significant reduction in burden, improved mental health, and an increase in well-being concerning the relation with the patient. Two studies evaluated the program for caregivers of youth with diverse mental health challenges, one quantitatively on a larger sample (*n* = 94) [28] and one qualitatively [29]. The results of the quantitative study showed a reduction in burden and grief and an improvement in coping strategies [28]. The results of the qualitative study showed that participants felt more able to manage their relatives’ mental health challenges, that the perceived quality of the relationship with their relatives improved, and that sharing caregiving experiences within a group was supportive [29].

Nevertheless, among the specific population of participants from families of people with BPD, the quantitative studies were conducted only on small samples.

The program has been implemented in several countries in Europe and a francophone section of the National Education Alliance for Borderline Personality Disorder (NEA-BPD) was organized in 2017 to disseminate the intervention. An initial implementation report of the French-speaking groups in Switzerland was published in 2016 [30]. Notwithstanding the current dissemination of the program, no quantitative study has been conducted thus far in French-Speaking Europe.

The aim of this study was to investigate implementation of the francophone version of the program on a large sample of families of people with BPD in Switzerland and France. Given that the main aims of the program are to reduce the psychological suffering of family members and teach new adaptive skills, we chose to explore the impact of the program on burden and depression, as in previous studies, furthering the exploration of changes in coping strategies and emotion regulation. We hypothesized that the suffering of participants (i.e., burden and depression) would decrease and that the resources of participants (i.e., coping and emotion regulation) would increase after completion of the program. A secondary hypothesis was that improvements in coping and emotion regulation resources would be associated with a decrease in burden.

## Methods

### Intervention

#### Intervention format

The FC program is manualized and structured into six modules of two sessions each (Table 1).

**Table 1:** Content of the FC program [23, 24]

Module 1:	Current information and research on BPD
Module 2:	The development of BPD, available treatments, comorbidity, emotional reactivity and dysregulation
Module 3:	Individual skills and relationship skills to promote participant emotional well-being • Emotion self-management • Mindfulness • Letting go of judgments • Decreasing vulnerability to negative emotions • Skills to decrease emotional reactivity
Module 4:	Family skills to improve the quality of family relationships and interactions • Letting go of blame and anger • Acceptance skills in relationships
Module 5:	Accurate and effective self-expression: how to validate
Module 6	Problem management skills • Defining problems effectively • Collaborative problem solving

All modules include specific practice exercises and homework [23]. The program is typically delivered weekly as a complementary intervention for families of people with BPD. In its original format [23], it is delivered only by family members who previously attended the program and received additional specific training. The program can also be part of an integrated framework of care in mental healthcare facilities, with the participation of professionals as co-leaders.

#### Intervention setting

The FC intervention was delivered in the traditional group setting initially established by its developers [23]. Leaders were trained by one of the official trainers of the NEA-BPD (Marie-Paule de Valdivia and Lynn Courey) and used a French translation of the official NEA-BPD FC manual [23]. Participation in the group was confirmed during an individual phone interview of the family member, performed by a former participant trained as a group leader in Versailles and Strasbourg or by a mental health professional in Geneva, Fribourg, and Montpellier. During this interview, the applying participant had to describe the difficulties they were having with their relative. The aim of the interview was to include caregivers of people presenting a diagnosis of BPD or features of severe emotion dysregulation in the program and to exclude participants whose relatives had a diagnosis or symptomatology highly evocative of a bipolar or schizophrenic disorder. In Fribourg and Montpellier, a small proportion of participants were also recruited via their relatives who were participating in a DBT program or who received specific individual care within a specialized clinic for BPD (with a diagnosis of BPD previously assessed using the Structured Clinical Interview for DSM-V (SCID-II) [31]). Participants attended one group session per week for three months (12 sessions in total).

In the Swiss centers (Geneva and Fribourg), groups were led solely by healthcare professionals (two psychologists in Fribourg, a psychologist and a nurse in Geneva). In Montpellier, they were also led mainly by healthcare professionals, with only certain specific sessions co-led by former participants. In Versailles and Strasbourg, groups were mainly co-led by trained former participants and a mental healthcare professional (psychiatrist or psychologist), with a few groups led solely by former participants.

### Design of the study

The study followed a pre-post observational design. Among a multicentric population of participants in the program in Switzerland and France between 2011 and 2020, we compared burden, grief, coping, and emotion regulation before and after the intervention. We used the Strengthening the Reporting of Observational Studies in Epidemiology (STROBE) guidelines to report our study [32]. Data were collected from participants of the FC program in five cities in Switzerland (Fribourg, Geneve) and France (Versailles, Montpellier, Strasbourg) between 2011 and 2020. Inclusion began at various time points depending on the implementation of the program in each center: 2011 in Fribourg, 2018 in Geneva, Versailles and Montpellier, and 2020 in Strasbourg. Participants were recruited to the program by various means: the relative’s clinician could directly propose that they participate or they could have learned about the program via the FC website or posters displayed in consultation settings. All participants were invited to complete paper forms containing an explanation of the study and several self-administered questionnaires. The level of burden, coping, grief, and emotion regulation were assessed at baseline (T1) and after the intervention (T2). Based on the observed reduction of the mean burden score of 22.7% after completion of the program in the study of Flynn et al. [25], we considered that participation in the program would lead to a decrease of 25% of the main burden score for the participants of our sample, as measured by the Involvement Evaluation Questionnaire (IEQ, see below). We assigned a baseline value for the IEQ for borderline caregivers of 33.54 based on the literature [33]. The sample size was then calculated with α = 0.05 and β = 0.05 for a one-sided paired comparison t-test before and after the intervention using the website https://biostatgv.sentiweb.fr. This resulted in a minimum sample size of 82 participants, each with data before and after the intervention.

### Measurements

Socio-demographic variables of the participants, their assessment of functioning and symptomatology of their relatives with emotion dysregulation, and their levels of burden, depression, emotion regulation and coping strategies were assessed through self-administered questionnaires at the beginning and end of the program.

#### Estimated intensity of symptoms and level of functioning of the relative with emotion dysregulation

At baseline, participants were asked to rate the intensity of their relative’s symptomatology and quality of functioning on two 10-point visual scales.

#### Age of symptoms onset of the relative with emotion dysregulation

Participants were asked to estimate since when difficulties with their relative had begun.

#### Burden

The burden of participants was assessed using the Involvement Evaluation Questionnaire (IEQ). The IEQ is a 31-item self-reported scale scored using a five-point Likert-scale. It was developed to evaluate the participants' experience of burden and the consequences of providing care to people with psychotic disorders [34]. It refers to the four previous weeks. Higher scores indicate a higher burden for the participant. The IEQ has been validated for caregivers of people with BPD and shows good psychometric properties, with Cronbach’s α ranging from 0.70 to 0.85, depending on the subscale considered [33].

#### Depression

Depression was assessed using the Center for Epidemiologic Studies-Depression Scale (CES-D). The CES-D is comprised of 20 items scored by the subject using a four-point Likert Scale. It has been widely used to assess depressive symptoms in community and population-based epidemiological studies [35]. The first validation study showed good internal consistency coefficients, between 0.85 and 0.90 in clinical and non-clinical samples [36]. The factor structure of the French translation was validated by Confirmatory Factor Analysis in 2011 [37].

#### Emotion regulation

Emotion regulation was assessed using the French version of The Difficulties in Emotion Regulation Scale (DERS). The DERS was first developed to identify difficulties in emotion regulation in six domains: lack of emotional clarity, non-acceptance of emotional responses, difficulties engaging in goal-directed behaviors, impulse control difficulties, lack of emotional awareness, and limited access to emotion regulation strategies. It is comprised of 36 items pertaining to the subject scored using a five-point Likert-Scale. A higher score indicates higher difficulties in emotion regulation. The DERS has been validated on two non-clinical samples, with Internal consistency of the original version reaching 0.93 (Cronbach’s α) [38]. The French version of the DERS has shown high congruence with the original version of 0.98 (Tucker’s phi), with an internal consistency of 0.92 (Cronbach’s α)[39].

#### Coping

Coping strategies of participants were assessed using the Family Coping Questionnaire (FCQ) [40]. It is comprised of 31 items about the past four weeks, scored using a five-point Likert scale from 1 = always to 5 = never. A higher score indicates better coping strategies.

Seven subscales can be distinguished (information gathering, positive communication, social involvement, coercion, avoidance, resignation, and the patient's social involvement).

It was first validated in Italian on caregivers of psychotic patients, with a Cronbach's alpha coefficient ranging from 0.68 to 0.83 [40]. It has since been validated in French [41].

#### Satisfaction

At the end of the program, participants were asked to evaluate, using a four-point scale, how much the intervention helped them in several domains corresponding to the modules of the program: level of information learned about the disorder (corresponding to modules 1 and 2), level of help in managing emotions (corresponding to modules 3 and 4), capacity to use existing resources (corresponding to module 6), and capacity to cope (corresponding to modules 3 to 6).

### Statistical analysis

1. **Sample analysis**
  a Flow-chart of the study
    We assessed the distribution of included participants between centers.
  b Sample characteristics at baseline
    We recorded the socio-demographic characteristics of the participants at baseline.
  c Group settings
    We recorded the proportion of the different group settings: groups led by professionals only, former participants only, or both, and the proportion of groups led by video-conferencing or face-to-face.
  d Comparison of samples between complete and incomplete datasets
    The main demographic characteristics and baseline levels of outcome measurements were compared at baseline between incomplete and complete data sets using Pearson’s chi-square tests, replaced by Fischer’s or Fischer-Freeman-Halton exact tests when the sample assumptions were not met for the chi-square tests, and an analysis of variance (ANOVA) for independent samples.
2. **Pre-post outcomes analysis**
  Measurements with 30% or more of the data missing were not considered. When less than 30% of the items of a scale were missing, the values were calculated by mean imputation. *P*-values < 0.05 were considered significant. To assess modifications of the scores of the outcome variables (burden, depression, emotion regulation, and coping) before and after intervention, we analyzed data at the multivariate and univariate level.
  a Multivariate analysis
    The four outcome variables moderately correlated with each other. Thus, to assess changes over time at the multivariate level, we performed a one-way repeated measures Multivariate Analysis of Variance (MANOVA), with the four outcome measurements (burden, depression, quality of coping strategies, and difficulties in emotion regulation) as dependent variables and time (measured before and after the intervention) as an independent variable.
  b Univariate analysis
    To assess changes over time at the univariate level, we performed separate paired Student t-tests for each outcome variable (burden, depression, quality of coping strategies, and difficulties in emotion regulation) before and after the intervention (T1, T2). Effect sizes were calculated using Cohen’s d: a value of 0.3 was considered small, 0.5 medium, and 0.8 strong.
3. **Influence of socio demographic features of participants and center on outcomes**
  We explored the potential influence of several sociodemographic factors on the outcome variables after completion of the program by assessing any interactions between sociodemographic variables and time (before and after the intervention). We performed two-way mixed ANOVAs for each socio-demographic variable. For each analysis, the within-subject factor was the outcome variable (IEQ, CESD, FCQ, DERS) and the between-subject factor was a sociodemographic factor (e.g., center or gender of the participant), whether binomial or polynomial. The following sociodemographic factors were explored: center, gender of the participants, gender of the relatives, relationship of the relatives to the participants, participants’ level of education, professional status of the participants, family situation of the participants, and presence of the relatives in the participants’ home. When a significant interaction was found, we performed a univariate ANOVA for each time point to assess the main group effect.
4. **Influence of changes in each coping strategy and difficulties in each domain of emotion regulation on burden reduction**
  Based on the transactional model, we hypothesized that an improvement in coping strategies and a decrease in emotion regulation difficulties after completion of the program would be associated with burden reduction. We chose to explore the influence of variations in each coping strategy and difficulties in each domain of emotion regulation on the burden using two separate linear regression models, one model for coping strategies (linear regression of the pre-post variation of the burden [IEQ] depending on the pre-post variation of the quality of each coping strategy [each FCQ subscale]) and one for emotion regulation difficulties (linear regression of the pre-post variation of burden [IEQ] depending on the pre-post variation of each emotion regulation difficulty [each DERS subscale]). We also included the intensity of the relative’s symptoms at baseline in each of the two linear analyses. We chose to use two separate linear regression models because the number of included subjects did not allow for a linear regression with all subscales of the FCQ and of DERS together in the same linear regression model.
5. **Satisfaction**
  We recorded the satisfaction after completion of the program.
  All analyses were carried out using IBM SPSS Statistics version 27.

### Ethical aspects

Participants were informed about the context and interest of the study. Participation was voluntary and refusal did not affect the participants’ involvement in the program. The study protocol was developed following the specific ethical local/national guidelines of each center (for the Réseau Fribourgeois de Santé Mentale: Comité éthique cantonal 016-REP-CER-FR; for Genève: the Ethics Committee of the Republic and Canton of Geneva; for the French centers: the Ethics Committee of Paris-Saclay University, CER-Paris-Saclay-2023- 046).

## Results

**Fig. 1:**
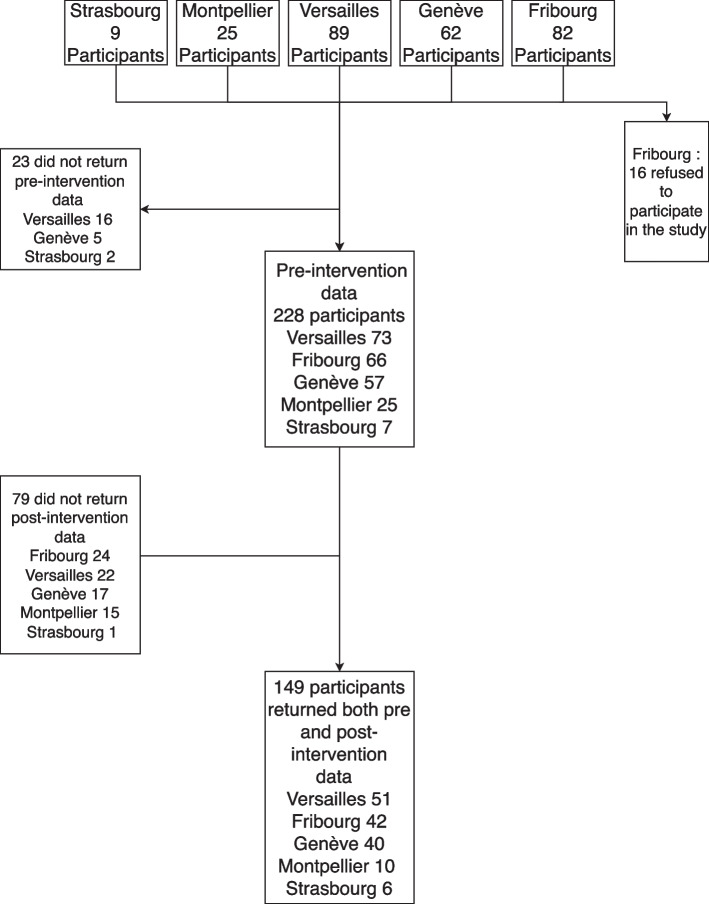
Flowchart of the study

**Fig. 2:**
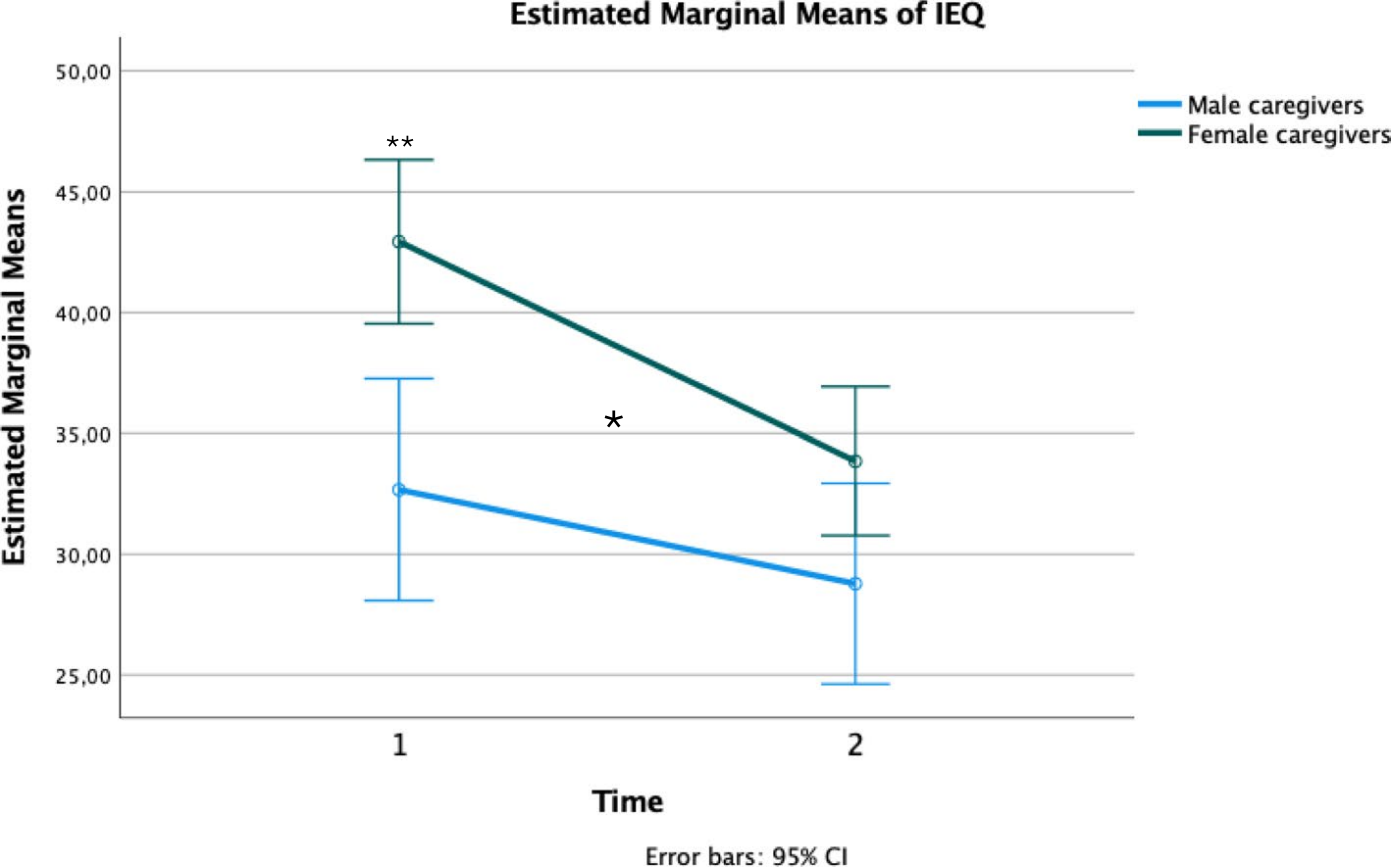
Effect of participant gender on the change in burden (IEQ). Two-way mixed ANOVA. *statistically significant interaction between time and gender, **statistically significant difference between the IEQ scores of men and women at T1

1. **Sample analysis**
  a Flow chart of the study
    In total, 267 participants attended the program: 123 in France (9 groups) and 144 in Switzerland (11 groups), resulting in a mean of 13.35 participants per group. Sixteen participants in Switzerland refused to complete the assessments. Twenty assessments were missing pre-intervention and 79 post-intervention. One group in Montpellier had to stop the intervention because of the COVID-19 pandemic, so no post-intervention assessment could be performed, representing incomplete data sets for 15 participants. In total, 149 participants completed both the pre- and post-intervention assessments (Fig. 1).
  b Sample characteristics at baseline
    Nearly two thirds of the participants were women (65.1%) and more than 3 of 4 was a father or mother (77.2%). The vast majority of participants had achieved post-secondary education (90.6%) and were working (67.8%). Relatives with emotion dysregulation were predominantly women (81.2%). Nearly half of the relatives were living with their caregiver (52.3%) (Table 2). The mean age of the participants was 53.31 years (SD 10.86) and the mean age of the relatives with emotion dysregulation was 26.01 years (SD 8.44). BPD symptoms had been noted by participants for more than six years (M=6.47, SD=5.74). Based on the reporting of the participants, the age of onset of symptoms was 19.6 years (*N*=141, SD=8.86) (Table 3).
  c Group settings
    In total, 55% of the groups were led solely by professionals, 30% by former participants and professionals together, 9% solely by former participants, and 7% mainly by professionals, with some sessions co-led with a former participant (see Additional File 1).
    Most of the groups were led fully face to face. In Versailles, groups switched to a video conference setting from March 2020 following restrictions due to the Covid-19 pandemic. One group had three of 12 sessions by videoconference and another group five. In Strasbourg, all sessions were held via videoconference. In total, 83% of participants received the intervention fully face to face, 13% with a few sessions via videoconference, and 4% completely via videoconference (Additional file 1).
  d Comparison of samples between complete and incomplete datasets
    In total, 79 of 228 included participants did not complete the T2 assessment, representing a rate of 34.7% of subjects with incomplete datasets. Nevertheless, almost all participants who did not fulfill the questionnaires completed the program (missing data were mainly due to organizational errors in the recall strategies to obtain the questionnaires from the participants). At baseline, these subjects did not differ significantly from the 149 subjects with complete datasets in terms of participant gender (X^2^ = 2.39, *p* = 0.12), the relative’s gender (X^2^ = 0.86 *p* = 0.35), the duration of symptoms (F = 0.11 *p* = 0.74), the age of the caregiver (F = 3.68, *p* = 0.06), the burden (IEQ: F = 2.03, *p* = 0.16), depression (CES-D: F = 0.79 *p* = 0.37), difficulties in emotion regulation (DERS: F = 0.93, *p* = 0.34), or coping (FCQ: F = 0.86, *p* = 0.35). We observed differences in the intensity of symptoms (F = 6.75 *p* = 0.01) and level of functioning (F = 4.11, *p* = 0.04), with participants with incomplete data showing a lower intensity of symptoms and a better level of functioning at baseline (Additional File 2, Additional file 3).
2. **Pre-post outcomes analysis**
  a One-way repeated measures MANOVA
    At the multivariate level, the four outcome measurements changed significantly over time (Within-subjects effect for Time: N = 144, Wilks’ Lambda = 0.703, F = 14.767, *p* < 0.001, partial η^2^ = 0.297). At the univariate level, all four outcome measurements changed significantly after the intervention (Within-subjects contrasts for Time, IEQ: *p* < 0.001, partial η^2^ = 0.188, CES-D: *p* < 0.001, partial η^2^ = 0.120, FCQ: *p* < 0.001, partial η^2^ = 0.218, DERS: *p* < 0.001, partial η^2^ = 0.090).
  b Paired student t-tests
    All four parameters improved significantly after the intervention (*p* < 0.0001), with a small effect size for depression (CES-D: d = -0.36) and difficulties in emotion regulation (DERS: d = -0.32) and a moderate effect size for burden (IEQ: d = -0.48) and coping (FCQ: d = 0.53) (Table 4).
3. **Influence of sociodemographic features of participants and center on outcomes**
  There was a significant interaction between time and the gender of the participant in terms of the decrease in burden, with the burden reduction being greater for women than men (F_1.145_ = 3.984, *p* = 0.048, η^2^ = 0.027). At baseline (T1), the burden was higher for women than men (F_1.146_ = 12.046, *p* < 0.001, partial η^2^ = 0.076). After the intervention (T2), there was no significant difference in the IEQ scores between male and female participants, although there was a significant trend (F_1.145_ = 3.757, *p* = 0.055, partial η^2^ = 0.025) (Fig. 2, Additional File 4). There was no other difference between subgroups of the participants based on sociodemographic features or center (the effect of the professional status of the participants on changes in coping could not be evaluated because the required conditions of variance homogeneity and covariance matrix equality were not respected).
4. **Influence of changes in each coping strategy and difficulties in each domain of emotion regulation on burden reduction**
  Among coping strategies, a decrease in coping by resignation significantly predicted a reduction in the burden (β = 0.19, t = 2.01, *p* = 0.047). Our model explained nearly 14% of the burden reduction (F = 3.791, *p* < 0.0001, adjusted *R* ^2^ = 0.138) (Table 5). Among emotion regulation strategies, the relative’s intensity of symptoms at baseline (T1) and improvement in emotional clarity predicted burden reduction (β = 0.22, t = 2.71, p = 0.008; β = 0.25, t = 2.82, p = 0.006, respectively). Our model explained nearly 10% of the change in burden reduction (F = 3.148, p = 0.004, adjusted R^2^ = 0.097) (Table 6).
5. **Satisfaction**
  When asked to rate how the program helped them, a vast majority of participants responded that the program “surely” helped them to learn about the disorder (89.5%) and how to better cope (84.6%). More than half of the participants replied that it “surely” helped to “learn to better manage [their] emotions” (53.1%) and to “better use existing resources” (53.8%). Nevertheless, for these two last items, 37.1% and 35.7% of participants, respectively, replied that they were only “probably” helped (Additional file 5).

**Table 2:** Participants’ characteristics

	N	%
**Center**		
Versailles	51	34.2%
Fribourg	42	28.2%
Geneva	40	26.8%
Montpellier	10	6.7%
Strasbourg	6	4.0%
**Gender of the caregiver**		
Woman	97	65.1%
Male	52	34.9%
**Relationship of the relative with emotion dysregulation to the caregiver**		
Spouse	18	12.1%
Father or mother	3	2.0%
Son or daughter	115	77.2%
Brother or sister	8	5.4%
Other	5	3.4%
**Level of education of the caregiver**		
Higher education	135	90.6%
Secondary or professional studies	14	9.4%
**Caregiver’s professional status**		
Employed	101	67.8%
Not active	46	30.9%
Missing data	2	1.3%
**Caregiver’s family situation**		
Does not live alone	114	76.5%
Lives alone	35	23.5%
**Gender of the relative with emotion dysregulation**		
Female	121	81.2%
Male	28	18,8%
**Presence of the relative in the caregiver's home**		
Yes	78	52.3%
No	70	47.0%
Missing data	1	0.7%

**Table 3:** Age of participants and relatives

	N	Minimum	Maximum	Average	Standard deviation
Age of the participant	149	24	82	53.31	10.86
Age of the relative	149	15	50	26.01	8.44
Duration of symptom evolution^a^	141	0.5	32	6.47	5.74
Age of symptoms onset^a^	141	5	48	19.6	8.86

^a^Relative with emotion dysregulation. Data in years

**Table 4:** Outcomes: univariate comparison

	T1		T2		Matched difference			Statistical test			
	M	SD	M	SD	M	SD	95% CI	t	df	*p*	d
IEQ	39.31	17.41	32.06	15.32	-7.25	15.23	[-9.73; -4.77]	-5.77	146	< 0.0001	-0.48
CESD	20.76	11.39	17.29	9.28	-3.47	9.72	[-5.05; -1.89]	-4.33	146	< 0.0001	-0.36
FCQ	3.57	0.45	3.77	0.42	0.20	0.37	[0.14;0.26]	6.38	145	< 0.0001	0.53
DERS	82.60	22.62	77.56	20.74	-5.03	15.79	[-7.60; -2.47]	-3.88	147	< 0.0001	-0.32

*IEQ* Involvement Evaluation Questionnaire, *CES-D* Center for Epidemiological Studies-Depression, *FCQ* Family Coping Questionnaire, *DERS* Difficulties in Emotion Regulation Scale, *SD* Standard deviation, *CI* Confidence interval, *df* Degrees of freedom, *p* Statistical significance, *d* Cohen’s effect size

**Table 5:** Linear Regression: Changes in burden depending on changes in the quality of coping strategies

	B	SE	β	t	*p*
Constant	-0.12	0.10		-1.18	0.241
Severity of the relative's symptoms at baseline	0.03	0.02	0.14	1.76	0.082
Δ Resignation	0.17	0.08	0.19	2.01	0.047*
Δ Personal interests	0.32	0.16	0.17	1.97	0.051
Δ Information	-0.21	0.13	-0.13	-1.64	0.103
Δ Positive Communication	-0.14	0.17	-0.07	-0.80	0.424
Δ Blame and coercion	0.07	0.14	0.04	0.51	0.610
Δ Avoidance	0.13	0.12	0.10	1.08	0.280
Δ Social involvement of the relative	-0.12	0.10	-0.11	-1.29	0.200

Enter method. Predicted variable: proportional changes in the IEQ: [IEQ (T1)—IEQ(T2)] / IEQ (T1)

*R* ^2^ = 0.187, adjusted *R* ^2^ = 0.138. B: non-standardized coefficient, β: standardized coefficient, t: test statistic, p: significance, * < 0.05

Δ: proportional change in the coping strategy considered: [strategy (T2)—strategy (T1)] / strategy (T1)

Significance of the model (ANOVA): F = 3.791, *p* < 0.0001

**Table 6:** Linear Regression: Change in burden depending on the change in emotion regulation skills

	B	SE	β	t	*p*
Constant	-0.14	0.10		-1.37	0.174
Severity of the relative’s symptoms at baseline	0.04	0.02	0.22	2.71	0.008*
Δ Lack of emotional clarity	0.37	0.13	0.25	2.82	0.006*
Δ Non-acceptance	0.10	0.10	0.08	0.95	0.344
Δ Difficulty with goal orientation	0.08	0.12	0.06	0.68	0.496
Δ Difficulty with impulse control	0.11	0.16	0.07	0.67	0.501
Δ Lack of emotional awareness	-0.12	0.13	-0.08	-0.94	0.349
Δ Limited access to regulatory strategies	-0.09	0.17	-0.05	-0.53	0.600

Enter method. Predicted variable: proportional change in IEQ: [IEQ(T1)-IEQ(T2)]/IEQ(T1)

*R* ^2^ = 0.142, adjusted *R* ^2^ = 0.097. B: non-standardized coefficient, β: standardized coefficient, t: statistical test, *p*: significance, * < 0.05

Δ: proportional change in the domain of emotion regulation difficulty considered: [difficulty (T1)-difficulty(T2)]/difficulty (T1). Significance of the model (ANOVA): F = 3.148, *p* = 0.004

## Discussion

This is the first large observational study conducted within the FC program in European French-speaking countries. Our first aim was to verify that the program helped participants to decrease their psychological suffering and increase their resources. Our secondary aim was to search for specific parameters that could influence burden reduction. We will first discuss the results related to our main outcomes. Then, we will examine the parameters that may influence burden reduction, namely the gender of the participants, improvement in coping strategies, and improvement in the understanding of one’s emotions. Finally, we will explore hypotheses related to specific elements of the program that could explain the observed reduction in depressive symptoms and improvement in emotion regulation strategies.

1. **Main outcomes: decrease in burden and depression and improvement in coping strategies and emotion regulation**

The main results of the study confirmed our expectations, showing that participants’ suffering decreased, namely their burden and depression, after completion of the program. In parallel, participants’ resources, such as coping strategies and emotion regulation, improved. These results are consistent with those of several previous studies on the program showing an improvement in burden, depression, grief, and mastery after completion [23–26, 28]. Moreover, when asked qualitatively how they thought the program had helped them, participants were clear about its usefulness in terms of learning about the disorder and how to better cope with the difficulties they face in their daily life with their relatives with emotion dysregulation. Learning about the disorder and finding new resources to cope are indeed the two objectives of psychoeducational interventions [42].

2. **Specific parameters that may influence a decrease in the burden**
  a Gender of the participants
    At baseline, the burden was higher for female than male participants. Moreover, the burden reduction was significantly greater among women. Previous studies on the FC program have also found a higher burden among female participants before the intervention concerning either the subjective component of the burden alone [24] or both the objective and subjective components [25]. In terms of the mental health of the participants, an extensive literature review highlighted contradictory results when searching for a difference in the burden based on gender [43]. The IEQ scale specifically focuses on the involvement of the participants with the relative. Expected gender-related social roles may account for the difference we observed in the burden at baseline. From a sociological perspective, caring for others is traditionally associated with feminine social roles [44]. According to the Caregiver Identity Theory, caregiving may emerge out of an existing role in a relationship, usually one that is familial [45]. In our study, mothers represented most of the participants. They may be more likely to involve themselves in caring for their relatives as a maternal duty. The FC program underlines the importance of taking care of oneself and setting self-boundaries regarding caregiving. This could be especially useful for mothers who might prioritize their relatives’ well-being over their own. A complementary explanation could be higher neurobiological vulnerability to stress-induced hyperarousal among women than men, as described in the literature [46].
  b Improvement in coping strategies
    We hypothesized that the decrease in burden is due both to the acquisition of better coping skills and an improvement in the caregiving relationship between the participant and the relative with emotion dysregulation. Our results show an increase in the quality of coping strategies within the caregiving relationship. During the FC program, participants learn problem-solving strategies and skills that are specifically focused on improving the caregiving relationship. For example, they learn how to assertively express their needs and boundaries, develop self-care, and validate the emotional experience of their relatives. Previous quantitative and qualitative studies on the program showed an increase in the feeling of knowing how to deal with the situation for participants [23–25, 28, 29], consistent with our results. In a recent conceptual framework of the informal caregiving burden, Gerain and Zech [47] outlined the roles of both coping skills and the quality of the caregiving relationship in their search for mediators of the caregivers’ burden. It is possible that the acquisition of better coping skills combined with an improvement in the caregiving relationship contribute to reducing the participants’ burden.
    An examination of the influence of the change in coping strategies on the burden of caregivers showed a decrease in coping by resignation to be related to burden reduction. Consistent with this result, previous studies on the FC program have shown an enhancement of mastery [23–25, 28] and a sense of being more able to cope with day-to-day difficulties [29]. Participants in our study had been struggling with their relatives’ symptoms for several years. For some, repeated failure in managing stressful situations with their relative may have led to learned helplessness [48], which can manifest as resignation. Developing strategies specifically designed to deal with the relative’s emotion dysregulation probably contributes to reducing such resignation. Being less resigned to the situation may lead participants to better engage in a meaningful relationship with their relatives, thus decreasing the burden. The decrease in coping by resignation may also be related to the decrease in observed depressive symptoms.
  c Improvement in the understanding of one’s emotions
    An increase in emotional clarity, in association with higher levels of BPD symptoms of the relative at baseline, was related to burden reduction. The association with BPD symptoms at baseline was an expected result according to the principle of regression to the mean. When participating in the program, an increase in emotional clarity is indeed expected, as one of the aims of the intervention is to develop a better understanding of one’s emotions. When practicing mindfulness, which is a central component of the program, participants are encouraged to note all their emotions without judgment. Existing empirical data suggest that group practice of mindfulness can contribute to improving emotional clarity. By being more attentive to physical cues and decreasing rumination processes, participants may become more aware of their own immediate intimate emotional experience [49].
    Concerning the influence of such increased emotional clarity on burden reduction, other empirical data suggest that individuals with a better capacity to identify their emotions may more efficiently apply problem-focused coping strategies and worry less [50, 51]. It is possible that when participants are more aware of the precise emotion they are feeling, it is easier for them to use the appropriate coping skill, thus reducing the burden.
3. **Aspects of the program that could account for the reduction in depression and improvement in emotion regulation**
  Here, we will focus on other specific elements of the program that could explain the improvements we observed concerning depression and emotion regulation.
  a Aspects of the program that could explain the reduction in depression
    i. Increasing acceptance and mindfulness
      Several aspects of the program could account for the reduction in depressive symptoms. First, DBT emphasizes the importance of acceptance in the process of healing. This is strengthened by the practice of mindfulness. The practice of both acceptance and mindfulness are key components of therapeutic interventions that have been proven to be useful in treating depression [52].
    ii. Improving understanding of the disorder and fostering hope
      Second, the program is designed to foster an understanding of the disorder and to develop a sense of hope that the disorder can be successfully treated. A qualitative study showed that participants experience more hope about the situation of their relative after the intervention. They also tend to be more able to take care of themselves, physically and emotionally [29]. These improvements in the subjective experience of the participants could all contribute to reduce depression.
  b Aspects of the program that could explain the improvement in emotion regulation
    i. Transactional improvement of emotion regulation within the family system
      Developmental models of BPD suggests that caregivers of borderline children may be prone to emotion dysregulation in their caregiving interaction [53, 54]. Specific components of the FC program are designed to enhance caregivers’ emotional well-being: learning how to set boundaries, taking time for oneself, paying attention to one’s own emotional needs, and setting priorities in the caregiving relationship. As outlined in the transactional model of emotion dysregulation of Fruzzetti et al. [10], improving emotion regulation of the caregivers is likely to lower emotional arousal for all family members via positive feedback loops, resulting in a virtuous circle for the entire family system.
    ii. Group format: feeling understood by other caregivers
      Among the possible mechanisms of change in the emotional state of the participants, we would like to highlight the group framework of the intervention. The FC program has been designed to be delivered from caregivers to caregivers [23]. Qualitative research on the program has shown that participants appreciate seeing their experiences as caregivers reflected in the experiences of other participants. Sharing similar emotions and stories appears to foster connections and a sense of belonging between participants [29]. Fonagy et al. have stressed the role of feeling understood in terms of one’s own experience as a way of facilitating learning, especially in the domain of communication skills [55]. Feeling validated in terms of one’s experience is a critical element of the basis of epistemic trust and the ability to be receptive to the teaching of new social skills. It is likely that participants can identify with facilitators, as they went through similar difficulties. Teaching from participants that have similar stories is likely more relevant from the perspective of the participants than the same skills delivered by a professional. This is in accordance with our qualitative results showing a very good level of participant satisfaction concerning learning about the disorder and how to cope better. Indeed, an important goal of the developers of the program was the idea of building a support network [23], and qualitative data shows that participants develop a sense of belonging when attending the program [29]. Increased social support is considered to be a key mediator in caregiver burden studies [47, 56, 57] and merits further exploration.

### Limitations

This study had several limitations. First, the observational before-after design did not allow demonstration of the efficacy of the program itself, as we did not have a control group. Future studies with a controlled design should be conducted to identify the specific effects of FC. Second, our research design did not include follow-up measurements. Retrospective recollection of such data would have been particularly difficult, as the inclusion period was spread out from 2011 to 2020. Third, concerning the evolution of the outcome measurements, we performed a comparison between before and after the intervention, without controlling for baseline levels. This would have allowed more accurate differentiation between individual outcomes. Fourth, we assumed that all observations were independent. However, within our sample, some participants were from the same family. We did not investigate how participants from the same family influence each other. Further studies could explore whether the effect of the intervention is stronger among participants of the same family and the responsible mechanisms. Fifth, not all centers contributed equally to the sample. The centers of Geneva, Fribourg, and Versailles accounted for most of our sample, as the program was implemented much later in Montpellier and Strasbourg. However, in subgroup analysis, we did not find any difference in terms of outcomes between centers. Finally, although the program aims to improve emotion regulation, we cannot rule out the possibility that the reduction of the burden may have had a positive effect on the emotion regulation of the participants. This could be explored using a longitudinal approach and path analysis in future studies.

## Conclusion

This study is the first to assess implementation of the FC program in European French-speaking countries. The results are encouraging and relied on a large sample of participants from five different centers over a long period. In this study, we show that the participants were highly satisfied with the program and that it resulted in an improvement in coping resources, a better ability to regulate their emotions, and a decrease in their burden and depression. The psychoeducational program is based on several complementary actions that aim to diminish the caregiver burden: provide knowledge about the disorder, acquisition of practical coping skills, strategies to improve the quality of family relationships, and building of a support network. Our study supports the relevance of integrating family psychoeducation in the care framework of people with BPD.

### Supplementary Information

Additional file 1. Group formats. Table describing who the group leaders were (health professionals, caregivers, both) and how the sessions were delivered (face to face or via video conferencing).

Additional file 2. Participants’ characteristics: comparison between complete and incomplete datasets. Table comparing complete and incomplete datasets at baseline of the participants’ socio-demographic characteristics.

Additional file 3. Pre-intervention outcome measurements: comparison between complete and incomplete datasets. Table comparing scores on the main outcome measurements at baseline between complete and incomplete datasets.

Additional file 4. Two-way mixed ANOVA (within-subject factor: IEQ, between-subject factor: gender). Table comparing estimated marginal means for the IEQ before and after completion of the program.

Additional file 5. Satisfaction of the participants. Qualitative measurements of participant satisfaction after completion of the program.

## Data Availability

The datasets used and/or analyzed during the current study are available from the corresponding author on reasonable request.

## Abbreviations

ANOVA: Analysis of variance
BPD: Borderline personality disorder
CAT: Cognitive Analytic Therapy
CES-D: Center for Epidemiologic Studies-Depression Scale
DBT: Dialectical Behavior Therapy
DERS: The Difficulties in Emotion Regulation Scale
FC: Family Connections®
FCQ: Family Coping Questionnaire
IEQ: Involvement Evaluation Questionnaire
MANOVA: Multivariate Analysis of Variance
MBT: Mentalization-Based Therapy
NEA-BPD: National Education Alliance for Borderline Personality Disorder
SCID-II: Structured Clinical Interview for DSM-V
STROBE: Strengthening the Reporting of Observational Studies in Epidemiology
T1: Pre-intervention time point
T2: Post intervention time point
