# Supplementary material for: Group intervention for family members of people with borderline personality disorder based on Dialectical Behavior Therapy: Implementation of the Family Connections® program in France and Switzerland
Source: Borderline Personal Disord Emot Dysregul. 2024 Jul 23;11:16. doi: 10.1186/s40479-024-00254-3 (PMC11265349; doi:10.1186/s40479-024-00254-3)
Supplement: Supplementary file 1 — Additional file 1. Group formats. Table describing who the group leaders were (health professionals, caregivers, both) and how the sessions were delivered (face to face or via video conferencing). [file 40479_2024_254_MOESM1_ESM.docx]

*Additional File 1. Group formats*

| GROUP LEADERS | N (participants) | % |
| --- | --- | --- |
| Both caregivers and professionals | 44 | 30% |
| Caregivers only | 13 | 9% |
| Mainly professionals | 10 | 7% |
| Professionals only | 82 | 55% |
| Total | 149 | 100% |
|  |  |  |
| GROUP DELIVERY | N (participants) | % |
| Videoconferencing only | 6 | 4% |
| 3 or 5 out of 12 sessions via videoconferencing | 19 | 13% |
| Fully face to face | 124 | 83% |
| Total | 149 | 100% |
