# Supplementary material for: Group intervention for family members of people with borderline personality disorder based on Dialectical Behavior Therapy: Implementation of the Family Connections® program in France and Switzerland
Source: Borderline Personal Disord Emot Dysregul. 2024 Jul 23;11:16. doi: 10.1186/s40479-024-00254-3 (PMC11265349; doi:10.1186/s40479-024-00254-3)
Supplement: Supplementary file 2 — Additional file 2. Participants’ characteristics: comparison between complete and incomplete datasets. Table comparing complete and incomplete datasets at baseline of the participants’ socio-demographic characteristics. [file 40479_2024_254_MOESM2_ESM.docx]

*Additional File 2. Participants’ characteristics: comparison between complete and incomplete datasets*

| Characteristic |  | Complete datasets | |  | Incomplete datasets | |  | Total | |  | Comparison | |
| --- | --- | --- | --- | --- | --- | --- | --- | --- | --- | --- | --- | --- |
|  |  | Average | N |  | Average | N |  | Average | N |  | F | p |
| Age of the participant |  | 53.31 | 149 |  | 49.94 | 77 |  | 52.16 | 226 |  | 4.78 | 0.03* |
| Age of the relative with emotion dysregulation |  | 26.01 | 149 |  | 28.56 | 78 |  | 26.89 | 227 |  | 3.68 | 0.06 |
| Duration of symptom evolution (in years) |  | 6.47 | 141 |  | 6.19 | 68 |  | 6.38 | 209 |  | 0.11 | 0.74 |
| Estimated intensity of symptoms of the relative with emotion dysregulation |  | 6.45 | 143 |  | 5.62 | 78 |  | 6.15 | 221 |  | 6.75 | 0.01* |
| Level of functioning of the relative with emotion dysregulation |  | 4.33 | 142 |  | 5.01 | 77 |  | 4.57 | 219 |  | 4.11 | 0.04* |

*Comparison: ANOVA. * p < 0.05*
