# Supplementary material for: Group intervention for family members of people with borderline personality disorder based on Dialectical Behavior Therapy: Implementation of the Family Connections® program in France and Switzerland
Source: Borderline Personal Disord Emot Dysregul. 2024 Jul 23;11:16. doi: 10.1186/s40479-024-00254-3 (PMC11265349; doi:10.1186/s40479-024-00254-3)
Supplement: Supplementary file 3 — Additional file 3. Pre-intervention outcome measurements: comparison between complete and incomplete datasets. Table comparing scores on the main outcome measurements at baseline between complete and incomplete datasets. [file 40479_2024_254_MOESM3_ESM.docx]

*Additional File 3. Pre-intervention outcome measurements: comparison between complete and incomplete datasets*

| Score |  | Complete data | | |  | Incomplete data | | |  | Total | | |  | Comparison | |
| --- | --- | --- | --- | --- | --- | --- | --- | --- | --- | --- | --- | --- | --- | --- | --- |
|  |  | Average | N | SD |  | Average | N | SD |  | Average | N | AND |  | F | p |
| IEQ (T1) |  | 39.19 | 148 | 17.41 |  | 35.74 | 79 | 17.22 |  | 37.99 | 227 | 17.39 |  | 2.03 | 0.16 |
| CES-D (T1) |  | 20.81 | 148 | 11.37 |  | 22.18 | 78 | 10.29 |  | 21.29 | 226 | 11.00 |  | 0.79 | 0.37 |
| FCQ (T1) |  | 3.57 | 147 | 0.45 |  | 3.51 | 76 | 0.41 |  | 3.55 | 223 | 0.43 |  | 0.86 | 0.35 |
| DERS (T1) |  | 82.51 | 149 | 22.56 |  | 85.44 | 77 | 19.60 |  | 83.51 | 226 | 21.60 |  | 0.93 | 0.34 |

*Comparison: ANOVA*
