# Supplementary material for: Group intervention for family members of people with borderline personality disorder based on Dialectical Behavior Therapy: Implementation of the Family Connections® program in France and Switzerland
Source: Borderline Personal Disord Emot Dysregul. 2024 Jul 23;11:16. doi: 10.1186/s40479-024-00254-3 (PMC11265349; doi:10.1186/s40479-024-00254-3)
Supplement: Supplementary file 4 — Additional file 4. Two-way mixed ANOVA (within-subject factor: IEQ, between-subject factor: gender). Table comparing estimated marginal means for the IEQ before and after completion of the program. [file 40479_2024_254_MOESM4_ESM.docx]

Additional File 4. Estimated marginal means of the IEQ before and after intervention

|  | Time |  | IEQ Estimated Marginal Mean |  | Standard. Error. |  | 95% Confidence Interval | |
| --- | --- | --- | --- | --- | --- | --- | --- | --- |
|  |  |  |  |  |  |  | Lower Bound | Upper Bound |
| Male participants | Pre-intervention (T1) |  | 32.68 |  | 2.32 |  | 28.08 | 37.27 |
|  | Post-intervention (T2) |  | 28.78 |  | 2.11 |  | 24.62 | 32.94 |
| Female participants | Pre-intervention (T1) |  | 42.94 |  | 1.72 |  | 39.54 | 46.34 |
|  | Post-intervention (T2) |  | 33.86 |  | 1.56 |  | 30.78 | 36.93 |

Two-way mixed ANOVA: within-subject factor: IEQ , between-subject factor: gender
