# Supplementary material for: Group intervention for family members of people with borderline personality disorder based on Dialectical Behavior Therapy: Implementation of the Family Connections® program in France and Switzerland
Source: Borderline Personal Disord Emot Dysregul. 2024 Jul 23;11:16. doi: 10.1186/s40479-024-00254-3 (PMC11265349; doi:10.1186/s40479-024-00254-3)
Supplement: Supplementary file 5 — Additional file 5. Satisfaction of the participants. Qualitative measurements of participant satisfaction after completion of the program. [file 40479_2024_254_MOESM5_ESM.docx]

*Additional file 5. Satisfaction of the participants*

| Thinks the program helped the participant to |  |  |  |  | | |  |  |  |  |  |  |
| --- | --- | --- | --- | --- | --- | --- | --- | --- | --- | --- | --- | --- |
|  |  |  |  | Surely |  | Probably |  | Perhaps |  | Not at all |  | Total |
| **Learn about the disorder** |  |  |  |  |  |  |  |  |  |  |  |  |
|  |  | N |  | 128 |  | 9 |  | 5 |  | 1 |  | 143 |
|  |  | % |  | 89.5 |  | 6.3 |  | 3.5 |  | 0.7 |  | 100 |
| **Learn how to cope better** |  |  |  |  |  |  |  |  |  |  |  |  |
|  |  | N |  | 121 |  | 18 |  | 3 |  | 1 |  | 143 |
|  |  | % |  | 84.6 |  | 12.6 |  | 2.1 |  | 0.7 |  | 100 |
| **Learn how to better manage their emotions** |  |  |  |  |  |  |  |  |  |  |  |  |
|  |  | N |  | 76 |  | 53 |  | 13 |  | 1 |  | 143 |
|  |  | % |  | 53.1 |  | 37.1 |  | 9.1 |  | 0.7 |  | 100 |
| **Learn how to better use existing resources** |  |  |  |  |  |  |  |  |  |  |  |  |
|  |  | N |  | 77 |  | 51 |  | 14 |  | 1 |  | 143 |
|  |  | % |  | 53.8 |  | 35.7 |  | 9.8 |  | 0.7 |  | 100 |
